# Recent developments in ultrasound approach for preservation of animal origin foods

**DOI:** 10.1016/j.ultsonch.2023.106676

**Published:** 2023-11-02

**Authors:** Akshay Rajendrabhai Bariya, Nikheel Bhojraj Rathod, Ajay Sureshbhai Patel, Jitendra Kumar Bhogilal Nayak, Rahul Chudaman Ranveer, Abeer Hashem, Elsayed Fathi Abd_Allah, Fatih Ozogul, Anet Režek Jambrak, João Miguel Rocha

**Affiliations:** a Department of Livestock Products Technology, College of Veterinary Science and Animal Husbandry, Kamdhenu University, Junagadh, Gujarat, India; b Post Graduate Institute of Post-Harvest Technology & Management, Roha, Raigad, Dr. Balasaheb Sawant Konkan Krishi Vidyapeeth, Maharashtra State, India; c Department of Veterinary Public Health and Epidemiology, College of Veterinary Science and Animal Husbandry, Kamdhenu University, Anand, Gujarat, India; d Botany and Microbiology Department, College of Science, King Saud University, P.O. Box. 2460, Riyadh 11451, Saudi Arabia; e Plant Production Department, College of Food and Agricultural Sciences, King Saud University, P.O. Box. 2460, Riyadh 11451, Saudi Arabia; f Department of Seafood Processing Technology, Faculty of Fisheries, Cukurova University, 01330 Adana, Turkey; g Biotechnology Research and Application Center, Cukurova University, 01330 Adana, Turkey; h Faculty of Food Technology and Biotechnology, University of Zagreb, Zagreb, Croatia; i Universidade Católica Portuguesa, CBQF - Centro de Biotecnologia e Química Fina – Laboratório Associado, Escola Superior de Biotecnologia, Rua Diogo Botelho 1327, 4169-005 Porto, Portugal; j LEPABE—Laboratory for Process Engineering, Environment, Biotechnology and Energy, Faculty of Engineering, University of Porto, Rua Dr. Roberto Frias, s/n, 4200-465 Porto, Portugal; k ALiCE—Associate Laboratory in Chemical Engineering, Faculty of Engineering, University of Porto, Rua Dr. Roberto Frias, s/n, 4200-465 Porto, Portugal

**Keywords:** Ultrasound, Livestock foods, Preservation, Non-thermal preservation

## Abstract

Ultrasound is a contemporary non-thermal technology that is currently being extensively evaluated for its potential to preserve highly perishable foods, while also contributing positively to the economy and environment. There has been a rise in the demand for food products that have undergone minimal processing or have been subjected to non-thermal techniques. Livestock-derived food products, such as meat, milk, eggs, and seafood, are widely recognized for their high nutritional value. These products are notably rich in proteins and quality fats, rendering them particularly vulnerable to oxidative and microbial spoilage. Ultrasound has exhibited significant antimicrobial properties, as well as the ability to deactivate enzymes and enhance mass transfer. The present review centers on the production and classification of ultrasound, as well as its recent implementation in the context of livestock-derived food products. The commercial applications, advantages, and limitations of the subject matter are also subject to scrutiny. The review indicated that ultrasound technology can be effectively utilized in food products derived from livestock, leading to favorable outcomes in terms of prolonging the shelf life of food while preserving its nutritional, functional, and sensory attributes. It is recommended that additional research be conducted to investigate the effects of ultrasound processing on nutrient bioavailability and extraction. The implementation of hurdle technology can effectively identify and mitigate the lower inactivation of certain microorganisms or vegetative cells.

## Highlights

• Ultrasound is a novel non-thermal technology for preservation of animal origin foods.
• This technology is known to inactivate enzymes and inhibit microorganisms extending shelf life.
• Some minor changes in sensory quality can be addressed by using as hurdle technology.

## 1 Introduction

The impact of food intake on economic, social, and political development is well acknowledged [1]. While, animal-derived foods offer superior nutrient quality and bioavailability compared to plant-based and other non-animal sources [2]. Furthermore, the consumption of animal-derived food products plays a significant role in promoting human health as they provide a rich source of essential nutrients [3]. With the increase in population, the demand for animal origin foods has increased due to their ability to provide high quality proteins having high biological value, fatty acids, minerals and vitamins. Combined production of animal origin foods from Beef, buffalo and poultry meat increased by 20 %, milk production increased by 24% and egg production increased by 30% during last ten years from 2011 to 2021 [4]. Furthermore, it was estimated that the demand for milk and egg is regarded to increase by around five and seven times respectively [5]. While in case of meat the consumption of beef, pork, poultry and sheep is projected to increase by 5.9, 13.1, 17.8 and 15.7 % respectively by 2030[6]. The demand and consumption of animal origin foods has increased globally with significant increase in per-capita consumption as noted in recent report of Henchion et al. [7]. Several studies have summarized several health benefits of animal origin foods such as their antioxidant, cardioprotective ability, ACE inhibitory, anti-inflammatory and prebiotic properties having positive impact on human health [8], [9], [10]. Animal source foods possess a varied nutrient composition and inherent conditions that render them conducive for the proliferation and development of spoilage microorganisms and food-borne pathogens [11]. Due to the high perishability of animal-originated foods, it is difficult to compete and survive in a dynamic global market without applying suitable processing and preservation techniques. Ensuring the consistent production and distribution of high-quality and safe products is of paramount importance in both local and global markets. Food-borne pathogenic organisms are responsible for a variety of major and challenging outbreaks in many countries. Certain enzymes may have an adverse impact on food quality [12]. Therefore, it is imperative to deactivate these enzymes to impede or avert their unfavourable effects. As per the Centers for Disease Control and Prevention (CDC), foodborne infections have a prevalence of affecting one-sixth of the population in the United States, leading to 1,28,000 hospitalizations and 3,000 fatalities annually [13]. It is estimated that foodborne illness will result in a financial burden of $35 billion annually, encompassing medical expenses, decreased productivity, and associated fatalities. Previous studies [14], [15], [16] have established a correlation between foodborne illness and the consumption of animal-derived foods such as poultry, milk, and fish. Conventional thermal techniques like pasteurization and sterilization are the most widely used methods for inactivating microorganisms in food and extending their shelf life [17], [18]. Consumers seek food with fresh-like qualities, high nutrient content along with retained sensory quality attributes [19]. However, these may not satisfy when food is treated with thermal processing because vitamins, taste, color, and other sensorial characteristics are reduced with thermal treatments [20], [21]. Nevertheless, it is often necessary to incorporate supplementary additives to enhance the quality of the products [22]. Various novel preservation techniques have been devised, which have the potential to eliminate microorganisms while significantly decreasing or eliminating the need for high temperatures, while simultaneously preserving the desirable qualities of freshness [20], [23]. Currently, there is an increasing awareness among consumers regarding the production processes involved in their food, leading to a greater inclination towards minimally processed food items. The food industry has shown interest in various non-thermal food processing techniques due to their ability to ensure optimal quality and safety of food products [24]. Non-thermal technologies possess the capacity to be employed in the realm of food processing, as they provide the opportunity for microbial and enzyme inactivation at ambient or sub-ambient temperatures [25]. In addition to their potential for reduced energy consumption and environmental sustainability, Picart-Palmade et al. [26] classify these technologies as either non-water-based or low-water-use. Ultrasound, being classified as non-thermal, exhibits promising potential as a viable substitute for conventional thermal food processing techniques [27]. The utilization of ultrasonic treatment in food preservation is a prevalent non-thermal method for heat-sensitive foods due to its ability to maintain sensory, nutritional, and functional attributes while enhancing shelf life and microbiological safety. As per the latest report published by Reports and Data, it is estimated that the worldwide market for food ultrasound will attain a valuation of USD 204.9 Million by the year 2028, exhibiting a noteworthy compound annual growth rate (CAGR) of 7.4% throughout the projected period. The market's expansion is predominantly propelled by a number of pivotal factors, such as the enforcement of rigorous regulations pertaining to food quality and safety, augmented investments by private entities in the food processing sector, an escalating desire for processed food items, and an intensified emphasis on curbing food wastage during the processing phases. The utilization of high-frequency sound waves in the food industry through ultrasound technology is of paramount importance as it deactivates microorganisms and enzymes, thereby preserving food while maintaining its quality standards [28], [29].

According to Yu and team [30], an ultrasound is defined as a sound wave with a frequency that exceeds the human hearing threshold of 20 kHz. Ultrasonic waves can be classified into two categories based on their frequency and intensity. These categories are low-frequency, which ranges from 20 to 100 kHz. According to Astráin-Redín et al. [31], there are two types of ultrasound: high-power ultrasound with intensity greater than 1 W/cm^2^ and low-power ultrasound with a frequency greater than 100 kHz and intensity less than 1 W/cm^2^. The relationship between sound intensity and sound frequency is one of inverse proportionality. Both types of ultrasound have demonstrated successful applications in various fields, particularly in the areas of food processing and safety-related industries [32]. According to Mohammed & Alhajhoj [33], the purpose of low-intensity applications is to disseminate energy across a medium for the purpose of transmitting information or gaining further insight into the properties of the medium. Diagnostic ultrasound, also known as low-intensity ultrasound, is commonly utilized as an analytical tool during quality control and processing phases. Its non-destructive inspection capabilities enable the determination of food concentration, viscosity, and composition [34]. High-intensity applications are deliberately designed to modify the properties of the propagation medium and are utilized in a diverse array of applications such as emulsification, defoaming, microstructure management, and textural attributes of fatty products. Additionally, they are employed in sonocrystallization and functional aspects of food proteins [35]. Food processing techniques are employed to alter the physical and chemical attributes of food [36]. This is achieved by subjecting the food to pressure, shear, and temperature variations in the medium through which it is conveyed [37]. The resulting cavitation effect leads to the elimination of bacteria in food products [22]. Most research on the utilization of high power in the food industry concentrates on techniques that transmit ultrasonic waves through a gaseous or liquid medium [38]. Ultrasound has been found to have multiple applications in the field of food processing. These applications include filtration, extraction, homogenization, drying, crystallization, defoaming, and meat tenderization. Additionally, ultrasound can be utilized as a preservation technique [39], [40], [41]. The efficacy of ultrasound as a preservative in food is attributed to its ability to deactivate microbes and enzymes [42]. Given the recent emergence of ultrasonication as a method for food processing and preservation, it is important to examine its implications and potential benefits. The focus of the current study was on the many methodologies utilized in the creation of ultrasound (US), the numerous types of generators, the mechanisms involved in microbial and enzyme inactivation by US, and the possible applications of these approaches in ensuring the safety of animal-based food products.

## 2 Generation of ultrasound and its application method

**Fig. 1:**
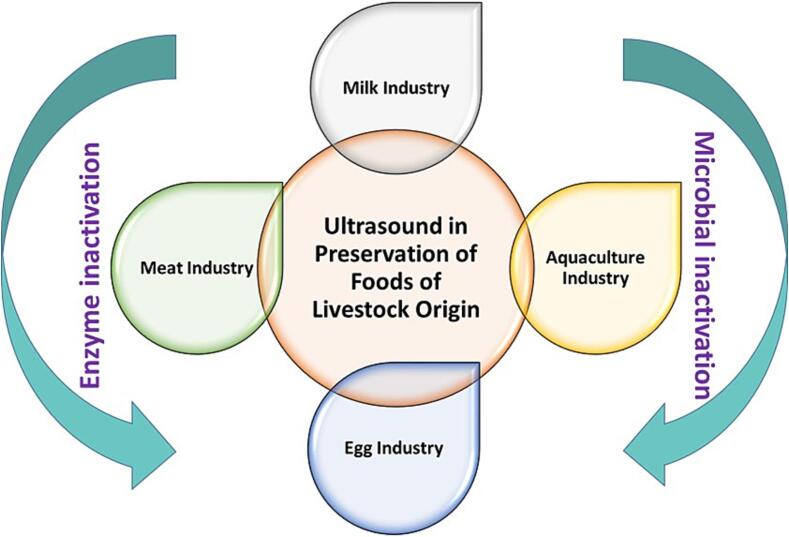
Impacts of ultrasound on preservation of foods of animal origin.

According to Mohammed, & Alhajhoj [33], an ultrasonic wave can be produced and transferred through the use of a device that includes an electrical generator, transducer, and sound emitter (reactor). The transducer in ultrasonic systems is driven by electrical energy provided by electrical generators. The ultrasound system produces a power rating that is suitable for its intended use. The system's power control is achieved indirectly through adjustments to the current (I) and voltage (V) settings. The production of ultrasonic waves in ultrasonic systems requires the transducer to undergo mechanical vibration, which converts mechanical or electrical energy from a generator into sound energy at ultrasonic frequencies [33]. The three fundamental types of transducers commonly employed in ultrasonic applications are piezoelectric, magnetostrictive, and fluid-driven transducers [33]. According to Yu and team [30], ultrasonic energy can be generated through the conversion of magnetic and electrical energy using piezoelectric and magnetostrictive transducers. According to Povey & Mason [43] research, fluid-driven transducers utilize mechanical energy to produce ultrasonic energy. The study compares the mechanisms of ultrasonic wave generation between magneto restraint transducers and piezoelectric transducers, which involve electroacoustic and acoustic-electric energy conversion, respectively. The primary role of an emitter is to transmit ultrasonic waves to the medium in a physical manner [38]. Horns and baths are the two most commonly used types of emitters [33]. The study also found that horns often require a sonotrode to be attached to the horn tip. According to Gallo and team [34], a variety of ultrasonic systems are accessible for food applications, based on the treatment material and desired impact. Achieving a satisfactory match between the application device and medium is crucial for transferring the maximum amount of acoustic energy to the treatment medium [31]. Ultrasound is a commonly used technique in the food industry, often applied through a liquid medium [31]. The experimental setup employed in the production and utilization of ultrasound is depicted in Fig. 1, which displays the Ultrasonic bath system and Ultrasonic probe system.

## 3 Types of Ultrasound

**Fig. 2:**
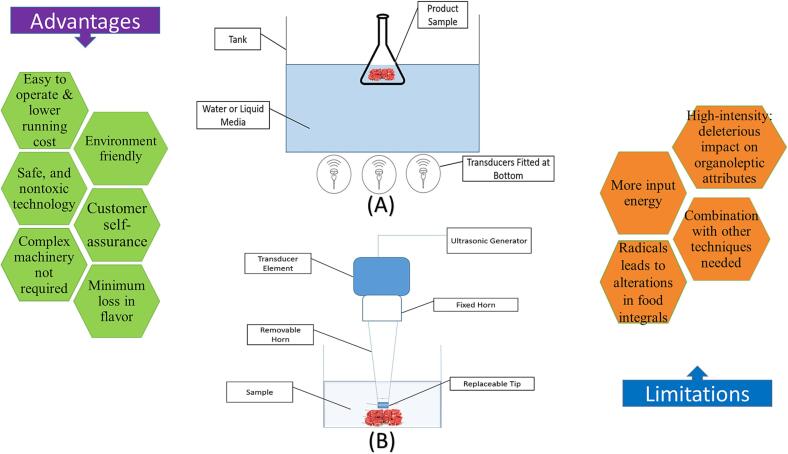
Ultrasonic generators A. Ultrasonic bath, B. Ultrasonic probe system.

The early utilization of ultrasonography on microorganisms was documented by Jacobs and Thornley[44]. The proposal was put up as a prospective technique for the sterilization of liquid food products. Another research suggested the use of ultrasound alone is not adequate for achieving the required reduction of microorganisms [45]. According to Chemat and team [46], the performance of ultrasound can be enhanced by utilizing it in conjunction with various food preservation techniques. Previous research has explored the combination of ultrasound with pressure, temperature, or both to enhance its effectiveness. This synergistic effect has been demonstrated in various studies [47], [48] as illustrated in Fig. 2.

### 3.1 Ultrasonication

Previous research have utilized ultrasonication at low temperatures [49]. The utilization of this approach is appropriate for the preservation of the nutritional composition of items that are sensitive to temperature, encompassing those that are susceptible to vitamin degradation, protein denaturation, amino acid deterioration, and non-enzymatic browning [49]. The process of inactivating stable enzymes and microorganisms in this particular application is characterized by a prolonged duration due to the low temperature utilized, resulting in substantial energy demands [47]. According to Zheng & Sun [50] research, the temperature increase during ultrasound application is influenced by the duration and intensity of ultrasonic power. Therefore, it is necessary to optimize the process to achieve the desired outcome.

### 3.2 Thermo sonication (TS)

The application of TS involves the concurrent use of ultrasound and heat modalities. The use of ultrasound and heat in combination can result in microbial inactivation comparable to traditional heat treatments [35]. Additionally, this approach can minimize operational demands and potential damage, such as reduced temperature or heat levels and shorter process times. Thermosonication has the potential to significantly reduce the time required for decimal reduction (D-value) [51]. Koshani and others [52] investigated the potential of combining ultrasound with low heat to reduce processing temperature and time. The study found that this technique resulted in a 16% reduction in processing temperature and a 55% reduction in processing time, which could make it more economically viable.

### 3.3 Mano-sonication (MS)

The MS technique involves the simultaneous application of ultrasound and pressure. The process involves the utilization of ultrasound technology in conjunction with mild pressures and low temperatures to deactivate enzymes and bacteria. According to Dolatowski [37], it has been found that the working efficiency of MS in deactivating enzymes and bacteria is higher than that of ultrasound alone, when both are subjected to the same temperature.

### 3.4 **Mano-thermo-sonication** (MTS)

MTS involves the application of heat, ultrasound, and pressure in a combined manner [47]. Particularly in those situations where thermotolerance is greater, MTS has proven to be a useful technique [53]. The application of MTS favors products sensitive to heat-induced deterioration [54]. Inactivation of multiple enzymes can be achieved in a shorter time frame compared to thermal treatments, without the use of low temperatures [46].

In a study conducted by Raso [55], the effects of various ultrasound types (MS and MTS) and heat on microbial inactivation were investigated using phase contrast microscopy. The results indicated that the heat treatment cells maintained their structural integrity, but the MTS treatment resulted in minimal disruption. Conversely, the MS treatment led to full destruction of the cells. The findings of the study suggest that ultrasound possesses the capability to inactivate microbial cells by causing the disintegration of their envelopes, which takes place in a binary fashion [56].

## 4 Microbial inactivation mechanism of ultrasound

**Fig. 3:**
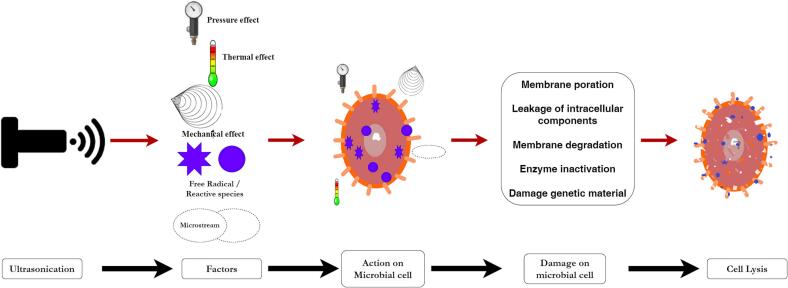
Proposed microbial inactivation mechanism of ultrasound.

The application of ultrasonic technology has been determined to be efficacious in the inactivation of microorganisms and the decontamination of food items. The aforementioned process is accomplished by inducing cavitation, fluctuating pressure, micro streaming creating shocks and the production of free radicals, as depicted in Fig. 3 [31], [57]. The literature typically documents two commonly recognized types of cavitation: hydrodynamic and acoustic [58]. The hydrodynamic and acoustic cavitation phenomena share similar underlying principles, differing only in their respective mechanisms for inducing localized pressure drops. The application of ultrasound in liquid induces acoustic cavitation, which is characterized by the generation, expansion, and subsequent collapse of bubbles. According to [59], when ultrasound travels through a medium, it generates a series of compression and rarefaction waves, similar to other types of sound waves. The medium experiences thermal, mechanical, and chemical effects due to the oscillation and collapse of bubbles caused by ultrasound propagation [60]. The expansion of bubbles is directly proportional to the increase in their surface area [61]. As the bubbles grow in size, the surrounding liquid medium loses its ability to absorb the gas present in the bubbles, causing them to continue expanding. The collapse of bubbles occurs when the energy of ultrasound and the fluctuation of the bubble wall are synchronized, resulting in their instability and violent collapse [61]. Also, the collapse of a cavity can result in a thermal phenomenon characterized by the emergence of localized hot spots at extremely high temperatures (10,000 °K) and pressures (1000 atm) [62]. While, a small amount of liquid is subjected to heat, resulting in rapid heat dispersion. However, the generated heat in the area is significantly high for a brief period of microseconds [63]. Mechanical phenomena such as shock waves, liquid microjets, and extreme shear forces can be produced under extreme circumstances [58], [64]. Acoustic cavitation generates microstreaming, which is a unique mechanism [65]. Micro Streams have the ability to generate a significant localized shear force, which can lead to the occurrence of substantial harm to microorganisms [66] furthermore, these substances have the potential to induce various forms of physical harm to the cellular membranes of the microbes. The cleavage of water molecules resulting in the formation of free radicals such as hydroxyl (HO) and hydrogen (H) radical is caused by the gas present in bubbles generated during implosion at high temperatures and pressures [58], [67]. The vulnerability of microorganisms to reactive species is increased due to the damage caused to their outer layer by heat and mechanical impacts [58]. Hydroxyl radicals have been identified as highly potent oxidants that can rapidly oxidize any species they encounter or interact with, leading to the formation of hydrogen peroxide (H_2_O_2_). The generated free radicals have an impact on the fluidity, permeability, and degradation of bacterial membranes. Additionally, when these free radicals penetrate the intracellular region, they inflict damage on internal components of microorganisms, resulting in cellular rupture. Moreover, the presence of free radicals can induce oxidative stress upon nucleic acids, leading to modifications in nitrogenous bases or disruption of the DNA double helix structure [68]. Ultrasound generates physical (pressure), chemical (free radicals), and thermal (heat) effects that impact the cell envelope [61]. These effects can lead to the disruption of cell walls and the release of intracellular content, ultimately resulting in cellular death. Bacterial cells lack a nuclear membrane, which distinguishes them from plant cells. This structural difference renders the genetic material of bacterial cells vulnerable to external factors such as pressure and temperature [69]. Hence, the primary factors contributing to microbial inactivation with ultrasonic treatment are the oxidation of intracellular amino acids, damage of the cell wall, and change of DNA material. The effectiveness of ultrasound treatment in terms of antimicrobial activity is influenced by various factors, including the duration and intensity of the ultrasonic treatment, hydrostatic pressure, temperature of the medium, type of microorganism, growth phase of microorganism, pH of the medium, water activities, and composition of the treatment medium [63], [70], [71].

The emergence of antimicrobial resistance in bacteria towards specific disinfectants is a critical and urgent matter of concern on a global scale. Previous studies have documented the utilization of ultrasound (US) to elicit a range of antimicrobial effects, potentially impeding the emergence of resistance [72], [73]. Furthermore, there is no evidence to suggest that the utilization of US technology in this particular situation is associated with the generation of any deleterious compounds or adverse effects on the sensory characteristics of the product.

## 5 Applications ultrasound in preservation of livestock products

The primary reason for food spoilage is the presence of microorganisms and the enzymatic activity [74], [75], [76], [77]. The level of inhibition and deactivation of microbial growth and enzymes is a determining factor in the selection of appropriate preservation methods [56], [78]. The application of ultrasound in food preservation has been studied due to its potential to inhibit microorganisms and prevent enzyme inactivation. This is attributed to the unique properties of ultrasound. Table 1 presents the effects of ultrasound when used in conjunction with other technologies for the preservation of livestock foods.

**Table 1:** Combination of ultrasound with other methods for preservation in food of animal origin.

**Product**	**Combination used with**	**Microorganisms**	**Treatment conditions**	**Reduction(logCFU)**	**References**
Meat					
Pork meat	Antimicrobial substance- Red wine, and yogurt marination	*L. monocytogenes, B. thermosphacta,* and *C. jejuni*	25 kHz and 300 W for 10 min	1-log reduction after 10 min	[149]
Chicken skin surface	1% aqueous lactic acid solution	*E. coli*	40 kHz and an intensity of 2.5 W/cm^2^ for 6 min	More than 1.5 log CFU/cm^2^	[150]
Skin and meat surfaces of pork	Pressurized steam(at 130 °C and a pressure of 3.5 to 5 atmospheres)	*Salmonella typhimurium, Salmonella derby, Salmonella infantis, Yersinia enterocolitica, and a non-pathogenic Escherichia coli*	30 to 40 kHz for 0.5 to 2 Second	mean reduction of 1.7 to 3.3 log CFU/cm^2^ on the skin surface and 1.1 to 2.5log CFU/cm^2^ on the meat surface	[151]
Broiler carcasses	Steam at 90–94 °C	*Campylobacter* and viable total count	30–40 kHz	reduced about 1.0 log CFU *Campylobacter* and 0.7 log CFU viable total count	[152]
Raw Poultry	Chemical- Trisodium phosphate (TSP), 2%, citric acid (CA) or 5 % capric acid sodium salt	*Campylobacter jejuni*	Ultrasonication at a frequency of 40, 60 and 80 kHz	Reduction of 4.5–4.6 log10 colony forming units CFU/cm^2^ at 80 kHz	[153]
		Total viable counts		Reduction of 1.9 log 10 CFU/cm^2^ at 80 kHz	
		*Enterobacteriaceae*		Reduction of 2 log 10 CFU/cm 2 at 80 kHz	
Hot dog sausages	–	Mesophilic, lactic acid and psychrotrophic bacteria	25 kHz, 200 W for 10.53 min at 74 °C	Reduction of higher than 3 log cycles in the count at end of storage	[154]
Beef slurry	Heat at 75 °C,	*C. perfringens spores*(NZRM 898 and NZRM 2621)	24 kHz, 0.33 W/g for 60 min	less than 1.5 log reduction for both *C. Perfringens* spores	[155]
Raw chicken meat	Plasma-activated Water	*Escherichia coli*	Frequency 40 kHz, intensity 9.6 W/cm^2^ for 0, 30, and 50 min	1.51 log_10_ CFU/mlreduction	[156]
		*Staphylococcus aureus*		0.85log_10_ CFU/ml reduction	
Beef	–	*Escherichia coli O157:H7*and *Bacillus cereus*	20.96 W/cm for 120 min	reduction percentage with around 40 %in both cultures	[157]
Chicken skin	Antimicrobial substance- Ethanol treatment- 30 %	*Salmonella typhimurium*	37 kHz and 380 W	>1.0 log CFU/g	[158]
Pork meat	Brine solutions	*Escherichia coli*	Ultrasonic Probe system with 20 kHz and a US bath with 33 kHz	Reduction of4 log 10 CFU/ml	[159]
Chicken meat and skin	Plasma-activated water	*E. coli* K12	40 Hz and an output power of 220 W at 40 °C for 60 min	Reduction up to 1.33 log CFU/ml	[160]
		*S. aureus*	40 Hz and an output power of 220 W at 40 °C for 60 min	Reduction up to 0.83 log CFU/ml	
Chicken skin	Peroxyacetic acid: 50–200 ppm	*S. Typhimurium*	37 kHz, 380 W, for 5 min	Reductions of 2.21 log CFU/g	[161]
		*C. jejuni*		Reductions of 2.08 log CFU/g	
Pork meat	–	*Bacillus cereus*	20 kHz, temperature of 70 °C for 13.56 min	0.47 CFU/g	[162]
Beef	Vacuum and modified atmosphere package	Mesophilic, Psychrophilic, *Staphylococcus*	High-intensity ultrasound with 40 kHz and 11 W/cm^2^for 60 min	average reduction of Mesophilic, Psychrophilic, *Staphylococcus,* bacteria1.17, 0.9, and 0.675 log10 CFU/ml respectively	[163]
Pork	–	*S. eyphimurium* and *E. coli*	20 KHz for 10, 20 and 30 min	1–4.3 and 1–4.6 log reduction in *S. typhimurium* and *E. coli* respectively	[164]
Raw meat emulsion	–	*Listeria monocytogenes*and *Lactobacillus delbrueckii*	400 W of power for 10 min	Inactivation values for *Listeria monocytogenes* and *Lactobacillus delbrueckii* were 63.3% and 53.4%, respectively	[165]

Milk					
Raw whole milk	Thermal	*Listeria innocua* ATCC51742	24 kHz; up to400 W,40–120 μm(0.86–2.85 W/cm^2^); 63 °C 0.5 °C; 2–30 min	5 log-reduction obtained	[166]
Raw milk	UV-C/thermal	Total aerobic mesophilic bacteria (TAMB), coliform bacteria (CB)	24 kHz; 240 W with UVlight of 13.2 W/cm^2^ for 15 min	4.79 log CFU/ml and 5.31 log-CFU/ml reduction for TAMB and CB	[93]
			24 kHz; 240 W at 65 °C for 30 min	3.29 log CFU/ml and 5.31 log-CFU/ml reduction for TAMB and CB	
Rennet cheese whey	Combination with heat pre-treatment	Total viable cells, coliform bacterial count, and viable yeasts and moulds count	24 kHz frequency, 400 W with exposure times 8 min	2.46,1.20 to1.79, and1.34 log cycles reduction of the total viable cells, coliform bacterial count, and viable yeasts and moulds count, respectively	[167]
Feta-type cheese	–	*Escherichia coli O157:H7, Staphylococcus aureus, Penicillium chrysogenum, or Clostridium sporogenes*	20, 40, and 60 kHz and intensity of 80% for 20 min	Sonication at 20, 40, and60 kHz reduced counts of *E. coli O157:H7*, *S. aureus*, *P. chrysogenum*, and *Cl. sporogenes* by 4.08, 4.17, and 4.28log; 1.10, 1.03, and 1.95 log; 1.11, 1.07, and 1.11 log; and 2.11, 2.03, and 2.17 log, respectively	[168]
Semi-skimmedsheep milk	–	Inactivation of total aerobic mesophilic bacteria, total coliform bacteria and *Staphylococcus*	Energy Density of 0.62–0.94 kJ/ mL, frequency of 20 kHz, 78 W for 6 and 8 min and 104 W for 4 and 6 min	Inactivation of total aerobicmesophilic bacteria, total coliform bacteria and *Staphylococcus*	[169]
Camel milk	–	Total aerobic bacteria, E. coli O157:H7 and *S. typhimurium*	20 kHz, 900 W for 15 min; energy density 8.10 kJ/ml	2 log cfu/ml reduction in total aerobic bacteria, Complete elimination of *E. coli* O157:H7 and a 4.4 log reduction in *S. typhimurium*	[170]
Milk	Combination with heat treatment	Total mesophilic aerobic bacteria (TMAB), total *Enterobacteriaceae* (TE),total coliform (TC), and total *Escherichia coli* counts	20 kHz work frequency as well as100% amplitude for30 and 40 min at 57.0 °C.	Around 2.12log cfu/mL mean reduction in TMAB, Complete complete inactivation of TE, TC, and total *Escherichia coli* counts	[171]
Raw milk	–	Total plate count (TPC) and Yeast and mold count (YM)	Power of 400 W for 8 min	Only 0.73 log reduction of TPC and 0.79 log reduction of YM	[172]
Raw bovine and camel milk	–	Total aerobic bacteria and coliforms bacteria	frequency of 20 kHz 170 W with 403 J and 210 W with 407 J energy	4 log cycles and total reduction of microbial load, complete disruption of coliforms bacteria	[173]

Egg					
Liquid whole egg	Heat	*Salmonella enteritidis*	40 W ultrasound at 50 °C for 5 min	Reduction of around 1 log	[174]
			40 W ultrasound at 55 °C for 5 min	Reduction of around 2.30 log	
Egg and eggshell	Heat	*Salmonella enteritidis*	24 kHz; 400 W, 60 μm; 54 °C; 5 min	Reduction of around 4.8 log	[175]
Japanese quail eggs	–	Aerobic microorganisms, coliform bacteria, *Salmonella* spp., *Staphylococcus* spp., and mold-yeast	35 kHz for 15 min	Reductions up to 20.7 %, 59.9%, 58.12 %, 61.60 % and 47.95 % for aerobic microorganisms, coliform bacteria, *Salmonella* spp., *Staphylococcus* spp., and mold-yeast, respectively, compared to control	[176]
Chicken egg	–	*E. coli*	140 W of power and 35 kHz for 30 min at 30 °C	Complete elimination of *E. coli* from contaminated eggs	[177]
Hatching and table eggs	Steam at 90 °C	*Salmonella* and *Enterobacteriaceae*	steam at 90 °C and ultrasound at 25–35 Hz for 1 sec.	complete reduction of *Salmonella* and *Enterobacteriaceae*	Musavian, 2018
Liquid whole egg	Lysozymeenzyme treatment	*S. typhimurium*	968 W/cm^2^ and 35 °C for 20 min	*S. typhimurium* reduction about 3.31log_10_cycleswith ultrasound and 4.26 log_10_ cycles with ultrasound and lysozyme combination	[178]
Whole Egg Liquid, EggYolk and Albumen	–	*E. coli*	40 kHz and 6.9 W absorbed power level at 60 min of treatment	Reduction of 0.5 log CFU/mL in whole egg liquid, 0.7 log CFU/mL in yolk and 0.5 log CFU/mL in albumen	[179]

Seafood					
Raw salmon fillets	Acidic electrolyzed water (AEW) and ultraviolet light (UV)	*L. monocytogenes*	45 kHz at a power of 200 W for 1 min	Reduction of 0.79 log CFU/g for (UV + US) and 0.75 (UV + US + AEW) log CFU/g	[180]
Sardines fish	Vacuum	Total mesophilic aerobic bacteria (TMAB), yeast mould and *Enterobacteriaceae*	20 kHz ultrasound at powers of 200 W/L, 300 W/L, and 500 W/L for 2 min	The 200-U group appears to be more effective than other groups at enhancing microbial quality.	[181]
*Tenualosa ilisha* fish fillets	Freezing	Total Plate Count	20 kHz and maximum power input about 1–5 W/cm^2^ for 10 min	More than 1 CF U/g (x10^4^) reduction than raw fish fillets	[182]
Smoked salmon	Heat	*L. monocytogenes*	20 kHz with 100% amplitude50°C for 5 min	Reduction of 2.44 CFU/ml	[183]
Sea bass fillets	Slightly acidic electrolyzed water	Total viable count	Frequency of 20 kHz, 600 W power for 10 min time	Reduction of 1.99 log_10_ CFU/g compared to control	[184]
Mackerel fillets	Plasma-activated water and peracetic acid	*Escherichia coli, Listeria innocua, and Pseudomonas fluorescens*	25 kHz at a power of 550 W for 10 min	Reduction of around 0.70 CFU/g *Escherichia coli*, 0.65 CFU/g *Listeria innocua* and 0.30 CFU/g *Pseudomonas fluorescens*	[125]
Grass carp	Plasma functionalizedwater and plasma functionalized buffer	*E. coli*	Frequency of 40 kHz and power level of 500 W	Reduction of 1.39 and1.31 log CFU/g*E. Coli*for ultrasound plasma functionalized Water and ultrasound plasma functionalized buffer, respectively.	[185]
		*S. putrefaciens*		Reduction of 1.49 and 1.39 log CFU/g *S. putrefaciens* for ultrasound plasma functionalized Water and ultrasound plasma functionalized buffer, respectively	
Thawed cod fillets	Hydration process	Total aerobic count, Mesophilic bacteria, Enterobacteriaceae, Proteolytic bacteria	Powers of 29.4 W/kg (100%), 14.7 W/kg (50%) and 2.9 W/kg (10%), for 20 min.	Reduced microbial growth compared to control	[186]
Mirror carp	Slightly acidic electrolyzed water	*Pseudomonas*	30 kHz for 5 min	Around 1. 7 log cfu/gm reduction compared to control at end of storage	[187]
Grass carp	Plasma functionalized buffer	*Escherichia coli and Listeria monocytogenes*	Frequency of 40 kHz and power level of 500 W for 15 min	Reduction of 3.92 log CFU/g for *E. coli* and 3.70 log CFU/g for *L. monocytogenes*	[188]
Silver Pomfret	Plasma functionalized Liquids and vacuum packaging	Total viable count, *Psedomonas and* yeast & molds	Frequency of 40 kHz, power level of 500 W and acoustic intensity of 5 min	Reductions of 1.99, 1.31, and 1.37 log CFU/g in Total viable count, *Psedomonas* and yeast & molds, respectively	[189]
Salmon	Blue light (BL) irradiation	*Vibrio parahaemolyticus*	Ultrasonic power at 25 KHz and 300 W for 15 min with BL at 216 J/cm^2^	Reduction of *V. parahaemolyticus* with the bactericidal rate of 98.81%.	[190]

### 5.1 Preservation of meat

The high moisture content, favorable pH, and nutrient-rich composition of meat render it susceptible to spoilage by microorganisms. The growth of spoilage and pathogenic bacteria is influenced by favorable conditions, which can result in the production of toxins, off-flavor, and discoloration [11], [79], [80]. Considering the great potential of ultrasound in the inactivation of microbes and enzymes it can also be used for improving meat quality [41]. The literature indicates that ultrasound waves have been found to have practical applications in the meat industry. In a study conducted by Pinon et al. [81], the effects of ultrasound application at varying power levels (20 kHz and 27.6 W/cm2; 40 kHz and 10.3 W/cm2; 850 kHz and 24.1 W/cm2) on the microbiological quality of chicken meat were investigated. The utilization of high power ultrasound with a frequency greater than 20 kHz and intensity of 27.6 W/cm2 has demonstrated outcomes that are comparable to other forms of treatment. The growth of lactic acid, mesophilic, and psychrophilic bacteria found in chicken meat was reduced by a high-intensity application. Suggesting application of US at 20 kHz and 27.6 W/cm2 for the preservation of chicken meat, based on their bactericidal property. Similarly, Caraveo and team [82] evaluated a high-intensity ultrasound (40 kHz) treatment on the physicochemical and microbiological characteristics of beef (*Semitendinosus* muscle). US application helped retain the normal pH relating to freshness value for beef muscle. Treatment (60 and 90 min.) reduced drip loss related to higher water holding capacity due to US treatment. The study observed a significant decrease in the levels of Total coliforms, Mesophilic, and Psychrophilic bacteria during a 10-day storage period. An inverse relationship was observed between the intensity of ultrasound (US) and the levels, as evidenced by a reduction in levels with an increase in US intensity when compared to the control sample. The study investigated the effect of high intensity ultrasound (US) on Total coliforms. Results showed a significant reduction of 3.05 and 3.52 Log CFU/ml in Total coliforms after US treatment for 60 and 90 min, respectively. The samples were stored for 10 days to assess the effectiveness of the treatment over time. During a 10-day storage period, the Mesophiles experienced a decrease of 1.04 and 1.68 Log CFU/ml, while the Psychrophiles experienced a decrease of 2.48 and 3.38 Log CFU/ml. Suggesting the ability of high-intensity US to destroy the microorganisms.

The combined effect of ultrasound (the US with frequencies of 25 and 130 kHz) with slightly acidic electrolyzed water (SAEW) for 10 min during the pre-chilling (10 °C) for chicken carcasses was reported [83]. According to their findings, the combination of US + SAEW significantly reduced the number of enterobacteria, mesophilic bacteria, lactic acid bacteria, and psychrotrophic bacteria. The study did not find any significant effect of the treatment on quality parameters such as lipid and protein oxidation, shear force, anaerobic glycolysis, and muscle structure. The study's findings suggest that the implementation of US + SAEW technology is a viable method for maintaining the quality of chicken carcasses in the pre-chilling phase. The processing of poultry meat comprises several stages, including the cooling process, which entails the immersion of carcasses in chilled water on a continuous basis. The identified step has the potential to cause cross-contamination. The research conducted by [84] sought to evaluate the efficacy of ultrasound as a standalone method or in conjunction with chlorine dioxide (ClO2) in eradicating *Salmonella typhimurium* (25 °C) and *Escherichia coli* (16 and 4 °C) in the water of poultry processing chiller tanks. The effects of varying ultrasound exposure durations (ranging from 1 to 9 min) on a fixed set of parameters (37 kHz frequency, 330 W power, and 25 °C temperature) using a bath. The experiment was conducted for a fixed duration of 1 min, during which the concentration of chlorine dioxide was adjusted to ensure a residual free chlorine level of 2.38 mg/L. Ultrasound treatment was found to reduce the activity of *Salmonella typhimurium* and *Escherichia coli* by 49% and 31%, respectively, according to the study's findings. The results of the study indicate that the presence of ClO_2_ did not lead to a decrease in activity for either microorganism when subjected to agitation. According to Smith [85], the application of low power ultrasonic treatment in combination with marination resulted in reduced microbial inactivation in broiler breast meat. The findings indicate that the incorporation of phosphate into the marinade led to a decrease in antimicrobial efficacy. The addition of low power US and phosphate to the marinade was found to have a significant impact on the inhibition of *Salmonella* and *Escherichia coli*. A recent study conducted by Vetchapitak [86] investigated the effectiveness of high power ultrasound (130 kHz at 1200 W for 15 min) in combination with chemical disinfectants (0.1% cetylpyridinium chloride and 0.01% sodium hypochlorite) for reducing *Campylobacter* on chicken carcasses. The study was conducted under both ambient and vacuum conditions for duration of 30 min. The application of Cetylpyridinium treatment under vacuum in combination with ultrasound resulted in a significant increase in the inhibition of *Campylobacter*, with a reduction of 0.94–1.64 log MPN/10 g observed. The application of chemical sanitizers to surfaces by the US has been found to enhance the effectiveness of treatments and lead to a decrease in bacterial load. In a recent study, Moazzami [87] investigated the potential impact of SonoSteam, a combination of ultrasound and steam, on naturally contaminated chicken carcasses. The study employed ultrasound technology with frequencies between 30 and 40 kHz and steam at temperatures of either 84 to 85 °C or 87 to 88 °C. The combination of ultrasound-steam treatment observed the average reductions of 0.5log CFU/g *C. jejuni*, 0.6 log CFU/g Enterobacteriaceae, 0.5 log CFU/g *E. coli*, and 0.4 log CFU/g for total aerobic bacteria. No significant variations in reduction were observed for any of the bacteria between the two distinct treatment temperatures, according to the research. Ultrasonic treatment has the potential to assist in the evaluation of the integration of mild and emergent technologies into the operational processes of the meat industry. Tables 2 present instances of the utilization of ultrasound treatment either alone or in conjunction with other methods to neutralize microorganisms and enzymes in diverse livestock-derived commodities.

**Table 2:** Combination of ultrasound with other methods for inactivation of enzymes in food of animal origin.

**Product**	**Combination used with**	**Enzyme**	**Treatment conditions**	**Status of Enzyme after treatment**	**References**
Meat					
Yellow-feathered chicken meat	Combination with heat treatment	Calpain and total proteases	Frequency of 40 kHz, 0.2 W/cm^2^ at 55 °C for 15 min	65.8% and 62.8%, decrease calpain and total proteases activity, respectively	[191]

Milk					
Whole milk	Combination with low heat treatment	Lactoperoxidase (LPO) and alkaline phosphatase (AP).	Amplitude level (0–80%) at Temperature (20 and 40 °C) for exposure times(30, 60, 90 and 120 s)	80% amplitude and 40°Cleads to inactivation of LPO:6.875 and AP: 3.813	[192]
Milk	–	β-galactosidase	Acoustic power of 20 W, and duty cycleof 10%	Only 25% activity loss	[193]
Full cream milk	–	Lactoperoxidase and alkaline phosphatase enzymes	750 W, 20 kHz, 24–26 °C; 2.5, 5, 6, 7.5, and 10 min	No inactivation of lactoperoxidase and alkaline phosphatase enzymes	[194]
Raw milk	–	Alkaline phosphatase	Amplitude of 70 and 100 for 50, 100, 200 and 300 sec	ALP activity not affected by any of the sonication treatments	[147]
Milk	–	Alkaline phosphatase	Amplitude of 91.2 μm and exposure time of 10 min	Only 5.2% reduction in the activity of alkaline phosphatase	[195]
Milk	Heat treatment	Plasmin activity	Frequency of 20 kHz, amplitude of 170 µm, 72 °C for 10, 30 and 60 s.	Reduction of 83 and 96% up to day 49 for both 30 and 60 s sonication times	[196]
Whole milk	Combination with heat treatment	Alkaline phosphatase activity	Amplitude (90%) for 20 min at 45 °C	Negative alkaline phosphatase activity	[171]
Raw milk	–	Alkaline phosphatase	Power of 400 W for 8 min	Only 14% reduction	[172]

Egg					
Egg white	Ultrasound with pressure (manothermosonication)	Lysozyme	117 µm,20 kHz and 200 kPa at 70 °C for 3.5 min	Decreased 10-fold lysozyme activity	[197]
Egg White Protein	–	Avidin activity	Power output of 400 Wfor16 min	avidin activity of the unprocessed sample reduce from 24.28 ± 0.52 μg/mL to around 16 μg/mL	[198]

Seafood					
Shrimp (*Pandalus borealis*)	Ultrasound-enzyme combination	Endo3 Enzyme	24-kHz frequency, 18.4-μm amplitude, 0.9-s pulse, 0.5% for 3 and 4 hr time at ≤ 5 °C temperature	Enzyme activity redcuce about 20% (from 61 to 49 U/mL) and 30% (from 61 to 43 U/mL) after 3-h and 4-h sonication, respectively	[199]

### 5.2 Preservation of milk

According to Górska-Warsewicz and others [88], milk and milk products are a rich source of nutrients, including high-quality proteins and essential micronutrients that are easily accessible. The high nutrient content of the substrate can facilitate microbial development, which may result in its degradation [89]. Spoilage is a widely recognized issue for the dairy industry due to the perishable nature of milk and its susceptibility to degradation by microbes and their enzymes [90]. Contamination of milk with pathogenic microorganisms has been identified as a potential cause of foodborne illness outbreaks. Milk is also known to contain enzymes produced by microbial contaminants and one prominent group is the extracellular substances produced by psychrotrophic microorganisms [29]. Considering the current status to sterilize the milk and milk products pasteurization is preferred [91], [92], however, exposing them to high temperature ranges nutritional composition and sensory quality reduced [93], [94]. As a result, there is growing interest in using novel non-thermal preservation techniques to preserve milk and milk products [95], [96]. These techniques may help to inactivate microorganisms and enzymes at lower temperatures than thermal pasteurization without compromising the products physicochemical, nutritional, and sensory qualities [97], [98], [99]. Considering the constraints ultrasound exhibits microbial and enzyme inactivation with preserved product qualities [29], [100], [101].

Impacts of thermosensation combined with pasteurization (72 °C for 15 s) and ultrasound (20 kHz at 150, 200, 300, and 400 W respectively for 10 min.) treatment on raw goat milk was studied [102]. Thermosonication treatment at power levels of 300 W and 400 W resulted in a reduction of the total bacterial count by greater than 2.08 log CFU/ml and 2.37 log CFU/ml, respectively, in raw goat milk. However, they also revealed that the same treatment had no impact on the color as well as soluble calcium and phosphorus contents in goat milk. Similarly, the effects of combined heat and ultrasound (temperature of 65 °C with average powers of 77, 104 and115 W at 20 kHz frequency) on total protease (plasmin and plasminogen) enzyme activity and quality (microbial and sensory) attributes of skim milk and cream [103]. The application of thermosonication at a power output of 115 W for a duration of 3 min resulted in a significant reduction of over 90% in the overall plasmin activity observed in fresh skim milk. Similar treatments had no negative impacts on sensory (off-aroma) quality. Raw milk treatment with non-thermal (100 W) and thermal (475 W) high-intensity ultrasound for microbial and enzymatic inactivation with different energy densities was studied Scudino and others [104]. The findings of the study indicate that the use of thermal treatment in combination with high-intensity ultrasound was effective in suppressing aerobic mesophilic heterotrophic bacteria (with a reduction of 3.9 logs). This effect was observed with an increase in energy density ranging from 1 to 7 kJ/ml. The study found that energy density levels above 3 kJ/ml resulted in the inactivation of alkaline phosphatase and lactoperoxidase activity.

Considering the advantages of US in preservation of milk and milk products, however there was doubt regarding the impacts on probiotic flora present exhibiting health benefits. It was reported that, ultrasound application at lower frequency, power and processing time improved viability of probiotic flora, with increased production having higher health benefits [105]. Specifically, US application on probiotic bacteria (lactobacilli and bifidobacteria) had no effects on viability at 45 °C and 9 pH [106]. Furthermore, lower intensity US was favorable for enhancing fermentation efficiency and reduce the time required when used at lag phase of bacterial growth suggesting regarding standardized process requirements to have no detrimental impacts on probiotic foods [107], [108].

### 5.3 Preservation of egg

The egg is a widely consumed food item that is recognized for its high-quality protein content, as well as its abundance of vitamins, trace minerals, and fatty acids. This has been noted in various studies [109], [110]. According to Réhault-Godbert and others [111], the egg has become a crucial component of the human diet due to its affordability and high digestibility. According to Sunwoo & Gujral [112], eggs exhibit a range of properties such as emulsifying, gelling, coloring, aromatic, and antioxidant properties. These multifunctional properties have made eggs a crucial ingredient in numerous food preparations. According to previous studies [113], [114], it has been found that while a fresh egg may be initially sterile, the eggshell can become contaminated by various microorganisms such as those found in fecal matter, nesting material, surrounding environment, and soil. The process of contamination involves the infiltration of microorganisms through the pores found on both the shell and inner membrane of the egg. These microorganisms then proceed to proliferate within both the egg white and yolk [115], [116]. According to previous studies [117], [118], it has been found that raw or minimally cooked egg products can serve as a potential source for the spread of food-borne diseases. Pasteurization is used to increase the shelf life of eggs and egg-related products while lowering consumer risks associated with pathogens that can be found in food, such as *Salmonella*. [119], the coagulation, foaming, and emulsifying properties of eggs are affected by pasteurization. Treatment with ultrasound could preserve the quality of eggs. Several studies have emphasized the potential applications of ultrasound in egg and egg products.

Ultrasonic treatment (140 W of power and 35 kHz frequency for 5, 15, and 30 min at 30 °C) with storage at different temperatures (10 d at 5 °C, and 10 d at 22 °C) on different quality parameters of egg were investigated by Sert and team [120]. Researchers found significant differences in total mesophilic aerobic bacteria (TMAB) values between treatments for albumen TMAB at the beginning and after storage for10 d at 5 °C and the lowest TMAB value in albumen (2.34 log cfu/g) and yolk (2.29 log cfu/g) was observed in samples treated by ultrasonic at 35 kHz for 30 min at 10 days storage at 5 °C. The study aimed to examine the impact of high-intensity ultrasound with varying durations (1, 5, 10, and 30 min) and parameters (20 kHz and 80% amplitude) on the eradication of *Salmonella enteritidis* in both liquid whole eggs and culture [121]. The study investigated the effect of different durations of high intensity ultrasound (US) on bacterial inhibition. The results showed that the bacterial inhibition observed after 1 min of US treatment (1.9 log CFU/ml) was similar to that observed after 5 min of treatment (2.2 log CFU/ml). Similarly, the bacterial inhibition observed after 10 min of treatment (3.6 log CFU/ml) was similar to that observed after 30 min of treatment (3.6 log CFU/ml). Further, the US application increased damage to *S. enteritidis* cells with an increase in exposure duration, scanning electron microscopy confirmed loss of structural integrity and deformation by high-intensity US exposure. The study's findings suggest that high-intensity ultrasound may be a viable method for quickly managing Salmonella in liquid whole eggs. This approach could serve as an alternative to traditional inactivation processes and be incorporated into hurdle strategies.

### 5.4 Preservation of seafood

The preservation of seafood is a critical process due to its highly perishable nature. Various techniques are employed to achieve this, including the use of chemical preservatives and the application of heat. These methods aim to enhance safety, extend the shelf life, and reduce the microbial load of seafood [122]. The utilization of certain methods is restricted due to their tendency to induce heat-related alterations, such as changes in flavor, texture, and appearance, particularly in fresh fish. Additionally, regulations concerning the application of chemical preservatives further complicate the matter. As per the findings of Bernardi et al. [123], maintaining freshness is a crucial aspect in ensuring the safety and quality of fish products. In recent years, non-thermal treatments such as ultrasound have become increasingly popular due to the growing demand for fresh and minimally processed food among consumers [25]. The current state of research on the use of ultrasound for seafood disinfection is in its early stages. Further research is necessary to enhance the industrial application of ultrasound. This involves optimizing parameters and examining the impact of ultrasound on the mass production of seafood products.

Pedrós-Garrido et al. [124] conducted a study to assess the impact of high-intensity ultrasound (30 kHz) at varying time intervals on the microbiological quality of various fish species (salmon, mackerel, cod, and hake) at the laboratory scale. A study was conducted to investigate the effects of ultrasound treatment on microbiological counts in oily fish species. The results showed a significant reduction in mesophilic and psychrophilic viable counts for salmon and mackerel, with reductions of up to 1.5 and 1.1 log CFU/g, respectively. However, white fish species only showed a reduction of 0.5 log CFU/g. The present study investigated the microbial reduction efficacy of white fish species, namely cod and hake, in comparison to other fish. The findings suggest that white fish exhibit lower reductions of microorganisms, which could be attributed to the surface's irregularity or roughness. This characteristic may provide bacteria with protection from ultrasound, thereby reducing the efficacy of microbial reduction.

**Fig. 4:**
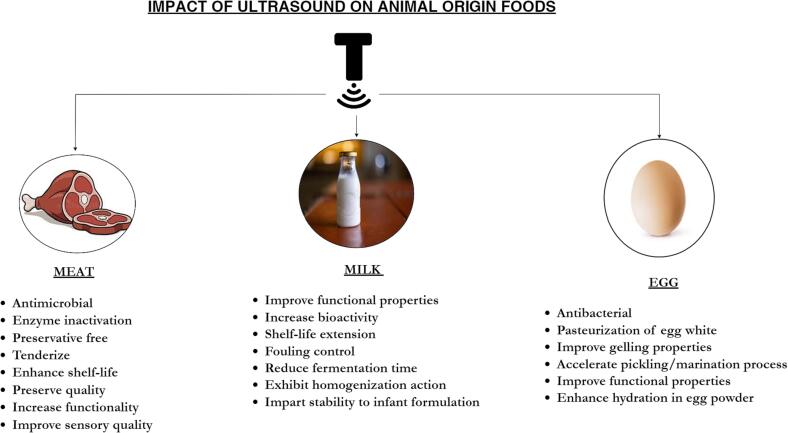
Overall impacts of US on animal origin foods.

The effectiveness of various decontamination methods (ultrasound, plasma activated water and paracetic acid) was investigated for raw mackerel fillets [125]. The methods included ultrasound (US) alone, plasma-activated water (PAW) alone, peracetic acid (PAA) alone, and combinations of these methods. The study evaluated the efficacy of these methods against both native microorganisms (total mesophilic bacteria—TMC and total psychrotrophic bacteria—TPC) and inoculated microorganisms (*Escherichia coli*, *Listeria innocua*, and *Pseudomonas fluorescens*). The research aimed to determine the most effective decontamination method for raw mackerel fillets. The present study investigated the efficacy of US alone or in combination with peracetic acid (PAA) and plasma-activated PAA for controlling total psychrotrophic and mesophilic counts in food samples. Results indicated that the application of US alone or in combination with PAA and plasma-activated PAA significantly improved the inhibition of total psychrotrophic counts (0.32, 0.38 and 0.70 log10 CFU/g) compared to total mesophilic counts (0.30, 0.27, 0.17 log10 CFU/g). A study was conducted to investigate the inhibitory effects of various treatments on *Listeria innocua*, *E. coli* K12, and *Pseudomonas fluorescens*. The results showed that treatment with the US in combination with peracetic acid, plasma-activated para acetic acid, and singly exhibited superior inhibition of the aforementioned bacteria. This was attributed to a synergistic effect between the treatments. Fig. 4 depicts the overall impacts of US on animal origin foods.

## 6 Factors influencing commercial implementation

The application of ultrasonic technology in food processing is widely acknowledged as a significant technique that can improve commercial efficiency and increase return on investment. Commercial-scale operations have the potential to achieve significant advancements in food product quality, process optimization, and cost reduction. Energetic optimization and heat recovery are two prominent areas that are gaining significant attention in the food industry. According to recent studies, one of the significant advancements in the field of ultrasonic equipment is the enhancement of its energy efficiency [126]. Additionally, the operational capacity of ultrasonic equipment has undergone progressive improvements over time [127]. The present development addresses the issue of internal heating, which is a major cause of system failure. According to previous studies [128], [129], the energy efficiency of existing ultrasound systems is estimated to be approximately 90–95%. This suggests that a significant proportion of the power transmitted to the transducer is effectively transferred to the medium. The industrial application of ultrasound technologies has been made possible by the advancement in equipment design and the development of efficient large-scale continuous flow-through systems [128]. According to previous studies [31], [128], the installation of a generator, cooling system, and other components into an existing system can be facilitated by factors such as improved efficiencies, lower costs, and simplified maintenance. Additionally, the appropriate sizing of these components can further enhance the ease of installation. According to Doona [129], soundproof cabinets have been developed to mitigate the noise generated by cavitation in ultrasonic processing. The energy requirement for material treatment per liter, commonly denoted as kWh/L, is similar to that of other unit operations in various industries. According to Kumar and others [130], the use of ultrasound technology has been found to result in lower energy consumption compared to traditional mechanical methods. One of the primary benefits of ultrasonic technology is its absence of mechanical components. Ultrasonic systems are distinguished by their lack of rotors, seals, grease, and other components. The sonotrode is a replaceable component that maintains direct contact with the treatment medium. According to Patist & Bates [128], the longevity of a sonotrode is subject to variation, ranging from one year to one and a half years. This variation is dependent on the amplitude and abrasiveness of the medium. The “Ultrafish” project, developed in partnership with the European Union, aims to improve the handling and processing methods of fresh and frozen fish products. Spanish company Scanfisk is leading the project, utilizing ultrasound technology to enhance existing techniques. The Ultrafish project conducted research on optimizing ultrasound technology for fish processing and observed promising results at the pilot plant level. Sanovo Technology Group headquartered located at Odense, Denmark acquired a SonoSteam technology from Danish independent technological service company FORCE Technology. SonoSteam technology utilizes a unique combination of steam and ultrasound delivered through specially designed nozzles, effectively eliminating bacteria. By harnessing the power of steam (90 °C) and ultrasound (frequencies ranging from 25 to 35 kHz) this innovative approach ensures the eradication of bacteria present in the treated area (Poultry Business, 2020). Other food companies like Faccenda Foods and Cargill have also installed a SonoSteam for disinfection of delicate food products such as poultry meat.

To the best of our understanding, there is currently no known federal legislation worldwide that specifically governs the use of US within the food business. The Food Safety Modernization Act (FSMA) has had a profound impact on the regulatory landscape of the food industry in the United States. The implementation of these legislation occurred in a sequential manner, commencing in the year 2016 and gradually being phased in over the course of multiple years. It is crucial to bear in mind that legislation and policies may vary across different countries and that they may have undergone revisions or alterations. The use of the Hazard Analysis and Critical Control Points (HACCP) and Hazard and Operability (HAZOP) principles is crucial when formulating a strategy for the production of an ultrasound-treated product. Furthermore, it is important to provide special attention to factors such as product handling, treatment conditions, and equipment hygiene [131]. Therefore, it is imperative for food manufacturers intending to employ US to establish communication and collaboration with regulatory agencies.

Process variations make ultrasonic industrial equipment and control system design difficult. To maximize this technology's potential, certain hurdles must be overcome [27]. US is an innovative technology that improves product quality, yet ultrasonic baths can present issues. The thermal action of ultrasound can cause the liquid medium to absorb heat, which may reduce its benefits in some applications [96], [132]. Continuous US can generate a lot of heat, which can affect food taste [35]. Uneven ultrasonic energy dispersion due to transducer-to-sample distance affects food thermal treatment uniformity. Tiny gas bubbles can affect ultrasonic wave transmission in some specimens. Use reflection measurements instead of transmission measures to resolve this issue, even with bubble signal interference [133]. Thermophysical factors including densities, compressibilities, heat capacities, and thermal conductivities affect ultrasonic prediction. Theoretical assessments for systems with several uncertain components are scarce. Ultrasonic properties may change when sample parameters are changed simultaneously. A simple sensor may not be enough to detect wide-ranging and difficult-to-address peaks. Temperature variations in a sample might cause errors when measuring temperature-dependent properties [134]. A techno-economic assessment is needed to understand, streamline, and implement this dynamic strategy. The “Ultrafish” project struggled. The main technological challenges of industrial ultrasonic processing includes maintaining high ultrasonic amplitudes in large horns, regulating transducer overheating during high-power operation, and addressing non-uniform treatment from flow-through reactor chambers bypassing the cavitation zone [135].

## 7 Advantages and limitations

The utilization of ultrasound in the food industry presents various benefits. The utilization of ultrasound technology in the industry is expected to provide a significant marketing benefit by enhancing customer confidence and creating a positive perception of both fresh and processed products. Ultrasound technologies have the potential to mitigate health and environmental risks associated with the production of carcinogenic halogenated by-products resulting from the use of chlorine-based chemical substances. According to previous studies [61], [136], the technology in question has been deemed environmentally friendly, safe, and nontoxic. The research findings indicate that the utilization of intricate machinery or a diverse array of technologies is not necessary. Ultrasound treatments have been found to be easy to operate and have been observed to contribute towards lower running costs, as well as efficient power output. According to previous studies [137], [138], ultrasound has been found to offer benefits over heat pasteurization, such as minimal flavor loss and notable energy conservation. In addition to its impact on preservation, this technique has potential applications in the food industry for various processes such as processing, extraction, emulsification, centrifugation, homogenization, and more.

While this technology offers numerous benefits, it also presents certain limitations. The use of high-intensity ultrasound in food processing has been found to generate heat, which can negatively affect the sensory and nutritional properties of the food product. Yusaf and Al-Jaburi [139] suggest that the adoption of ultrasound technology on a commercial and business scale requires consideration of the increased input energy demands associated with its use. According to X. Li & Farid [140], the effectiveness of ultrasound in preventing microbial and enzymatic inactivation has been inconsistent. The potential for inactivation may arise due to the presence of various obstacles, including the synergistic effect. The utilization of ultrasound results in the production of radicals due to the critical temperature and pressure conditions, leading to modifications in food components. The accumulation of radicals (OH and H) on the surface of cavitation bubbles leads to the initiation of radical chain reactions [141]. These reactions ultimately result in the production of degradation products and various quality issues in the final product.

## 8 Impacts of US on sensory aspects of animal origin foods

Sensory aspects are important characteristics related with actual quality perceived by the consumer, which is important for novel food processing technologies which results in constant new product development [142], [143]. US application in meat has been reported to enhance the sensory quality [144]. Application of US (250–750 W) on unsomked bacon ripened for 10 days was evaluated [145]. Application of US at 500 W level was found to improve sensory quality, while further increase in power to 750 W increased saltiness perception attributed to impacts of higher cavitations. In case of jumbo squid optimized US (186.9 W) application resulted in enhanced sensory quality which was superior in comparison with control [146]. In milk ultrasonication (200 W) for 2 min was found to have acceptable sensory qualities in comparison to control samples [147]. Increase in off-flavour development with increase in intensity of US from 100 to 400 W for longer duration was observed [138]. Recently, Scudino et al. [99] evaluated role of ultrasound application on cheese (Minas Frescal) acceptability. Amongst the attitude evaluation, the nutritional and sensorial value of the product should be preserved received maximum score. While, cheese made with high intensity ultrasound was liked by good number of consumers [99]. On the contrary, high intensity ultrasound (400 W) resulted in weaker taste, increase sourness with increase in storage. Imparted foreign taste and odour with increase in power intensity of US [148].

## 9 Conclusions

The present apprehensions around instances of food-borne epidemics have emphasized the imperative of ensuring the production of food that is safe for consumption by individuals. In over a decade, there has been a notable augmentation in the purchasing power of consumers, accompanied by a concomitant elevation in their level of discernment. Consumers seek food products that possess a high concentration of health-enhancing components, while simultaneously safeguarding nutritional integrity and being free from any traces of chemical residues. This is a significant obstacle for the food business and emphasizes the necessity of advancing innovative decontamination technology. The utilization of ultrasound in many applications can be regarded as a safe, non-toxic, and environmentally sustainable technique for ensuring food safety and maintaining high standards of quality. Ultrasonic cavitation induces both physical and chemical stresses, which therefore cause substantial harm to the cellular membranes of microorganisms, ultimately leading to their demise and the deactivation of enzymes. Ultrasound possesses the capability to be employed in both the processing line and for products, hence simplifying the expeditious execution of certain procedures. In addition, processors would also see benefits from an extension of the product's shelf life. This would enable longer storage and stability durations, hence facilitating access to geographically remote markets. Ultrasonic techniques have demonstrated efficacy in preserving the physical and sanitary attributes of food goods without causing any detrimental effects. When utilized in a suitable manner, it has the ability to substitute conventional sanitation techniques without compromising the sensory attributes of food products. Nevertheless, there are still several unresolved issues that need to be investigated in order to enhance the efficacy of this technology across different industries. Ultrasound cavitation has garnered attention across multiple fields pertaining to food quality and processing, encompassing non-destructive quality assessment, homogenization, modification of food constituents, microbial eradication, enzyme deactivation, and other related applications. The aforementioned applications require a minimal quantity of ultrasonic energy, a scalable method that leads to substantial cost reductions in processing. However, even complying with existing food regulatory requirements, relying exclusively on ultrasound may not always be sufficient to completely eliminate microbiological contamination. In order to guarantee the microbiological safety of animal-derived food, it is imperative to employ ultrasound treatment in conjunction with other decontamination techniques, since the sole use of ultrasound treatment is insufficient to achieve adequate decontamination. At present, there is a lack of established laws pertaining to the application of ultrasonication in the context of food processing. The use of ultrasonication necessitates a collaborative effort between food processors and regulatory bodies. Likewise, it is imperative to emphasize the significance of product handling, treatment parameters, and equipment hygiene. A techno-economic review is required in order to streamline and implement this dynamic process.

## 10 Future prospects

Numerous investigations have been carried out in the field of food technology utilizing ultrasound technologies. Nevertheless, further research is required to develop automated ultrasound systems that can be implemented in industrial settings, resulting in reduced labor, cost, and energy consumption, while simultaneously ensuring the safety of food products derived from livestock. Moreover, an enhanced comprehension of the intricate principles that underlie the activities and impacts of ultrasound on food qualities could potentially bolster the future applications of ultrasound in the food industry. The integration of ultrasound with other techniques yields favorable outcomes in the context of the comprehensive quality of the product, thereby warranting further investigation. Further advancement in the industrial utilization of ultrasound requires the refinement of process parameters and research tailored towards industry-specific evaluation of the effects of acoustic treatment on food production at scale. The livestock feed industry has highlighted the need to establish optimal parameters, dosages, and treatment combinations, as well as to improve the machinery's capabilities, in order to advance the commercialization of ultrasound technology.

## Funding

The authors would like to extend their sincere appreciation to the Researchers Supporting Project Number (RSP2023R134), 10.13039/501100002383King Saud University, Riyadh, Saudi Arabia.

## CRediT authorship contribution statement

**Akshay Rajendrabhai Bariya:** Conceptualization, Writing – original draft, Writing – review & editing. **Nikheel Bhojraj Rathod:** Conceptualization, Writing – original draft, Writing – review & editing. **Ajay Sureshbhai Patel:** Writing – original draft. **Jitendra Kumar Bhogilal Nayak:** Writing – original draft. **Rahul Chudaman Ranveer:** Writing – original draft. **Abeer Hashem:** Writing – review & editing, Funding acquisition. **Elsayed Fathi Abd_Allah:** Writing – review & editing, Funding acquisition. **Fatih Ozogul:** Conceptualization, Writing – review & editing. **Anet Režek Jambrak:** Writing – review & editing. **João Miguel Rocha:** Writing – review & editing.

## Declaration of Competing Interest

The authors declare that they have no known competing financial interests or personal relationships that could have appeared to influence the work reported in this paper.

## Data Availability

No data was used for the research described in the article.
